# Research, education and capacity building priorities for violence, abuse and mental health in low- and middle-income countries: an international qualitative survey

**DOI:** 10.1007/s00127-021-02061-5

**Published:** 2021-03-25

**Authors:** Roxanne C. Keynejad, Abigail Bentley, Urvita Bhatia, Oliva Nalwadda, Fikru Debebe Mekonnen, Parveen A. Ali, Julie McGarry

**Affiliations:** 1 Department of Health Service and Population Research, King’s College London, London, UK; 2 London School of Hygiene and Tropical Medicine, London, UK; 3 Sangath Addictions Research Group, Porvorim, Goa India; 4 Butabika National Referral Hospital, Kampala, Uganda; 5 Edna Adan University Faculty of Public Health, Hargeisa, Somalia Somaliland; 6 University of Sheffield Health Sciences School, Sheffield, UK; 7 University of Nottingham School of Health Sciences, Nottingham, UK

**Keywords:** Violence, Abuse, Mental health, Global mental health, Low- and middle-income countries, Capacity building

## Abstract

**Purpose:**

Despite the World Health Organization and United Nations recognising violence, abuse and mental health as public health priorities, their intersection is under-studied in low- and middle-income countries (LMICs). International violence, abuse and mental health network (iVAMHN) members recognised the need to identify barriers and priorities to develop this field.

**Methods:**

Informed by collaborative discussion between iVAMHN members, we conducted a pilot study using an online survey to identify research, education and capacity building priorities for violence, abuse and mental health in LMICs. We analysed free-text responses using thematic analysis.

**Results:**

35 senior (29%) and junior researchers (29%), non-government or voluntary sector staff (18%), health workers (11%), students (11%) and administrators (3%) completed the survey. Respondents worked in 24 LMICs, with 20% working in more than one country. Seventy-four percent of respondents worked in sub-Saharan Africa, 37% in Asia and smaller proportions in Latin America, Eastern Europe and the Middle East. Respondents described training, human resource, funding and sensitivity-related barriers to researching violence, abuse and mental health in LMICs and recommended a range of actions to build capacity, streamline research pathways, increase efficiency and foster collaborations and co-production.

**Conclusion:**

The intersection between violence, abuse and mental health in LMICs is a priority for individuals with a range of expertise across health, social care and the voluntary sector. There is interest in and support for building a strong network of parties engaged in research, service evaluation, training and education in this field. Networks like iVAMHN can act as hubs, bringing together diverse stakeholders for collaboration, co-production and mutually beneficial exchange of knowledge and skills.

**Supplementary Information:**

The online version contains supplementary material available at 10.1007/s00127-021-02061-5.

## Introduction

Gender-based violence (GBV) refers to acts of violence perpetrated against a person’s will, based on gender norms and unequal power relationships, causing harm to women, girls, men and boys [1]. Non-partner sexual violence and intimate partner violence (IPV) are common forms of GBV. IPV refers to behaviour by a partner or ex-partner, which causes physical, sexual or psychological harm [2], while domestic violence (DV) is also perpetrated by non-partner family members [3]. GBV is highly prevalent, including in low- and middle-income countries (LMICs)[4, 5] and an important social determinant of health [6]. The World Health Organization (WHO) [7], United Nations[8] and World Psychiatric Association[9–11] have prioritised preventing and addressing GBV, its physical and mental health impacts.

The relationship between GBV and mental health is bidirectional, with GBV increasing the risk of mental disorders, which in turn increase vulnerability to GBV. For example, IPV is associated with subsequent depressive symptoms, suicide attempts [12] and alcohol use disorders [13], which predict later IPV. Investigating the mental health impacts of GBV is a research priority, especially for LMICs [14]. There is growing evidence for psychosocial interventions for mental health in LMICs [15]. However, a recent meta-analysis investigating the impact of IPV exposure on psychological interventions’ effectiveness [16] identified just two RCTs tailored for women experiencing IPV in LMICs [17, 18]. The role of violence and abuse in mental health and treatment response in LMICs, therefore, requires much greater research attention [19, 20].

In 2011, research priorities for mental health and psychosocial support in humanitarian settings were identified through a consensus-based approach with 82 academics, policy makers and practitioners [21]. Priorities included investigating stressors, optimal assessment, monitoring and intervention approaches for humanitarian settings. In 2012, a Delphi study surveyed 65 members of the preventing violence across the lifespan (PreVAiL) research network [22]. Respondents prioritised mediators and moderators of the relationship between violence exposure and mental health outcomes, but were based exclusively in HICs. Child health and nutrition research initiative methodology was used to identify research priorities for adolescent health in LMICs [23]. Beginning with 512 research questions proposed by 142 experts, the ten most prioritised questions across all health areas addressed integration of health services and platforms for vulnerable populations (including GBV survivors and young people with mental health problems). There are no published studies of education or capacity building priorities in the field of violence, abuse and mental health in LMICs.

There is increasing recognition that global health initiatives funded or led by HIC institutions must build research capacity in LMICs [24]. Calls have been made for active leadership from LMICs and partnership approaches to HIC involvement, to address power imbalances in global health research and practice [25]. However, studies of the capacity building needs of early-career researchers in LMICs and programmes designed to address them are limited [26]. AFFIRM was a five-year project incorporating Master's degree fellowships, doctoral funding and research short courses for capacity building in Ethiopia, Ghana, Malawi, South Africa, Uganda and Zimbabwe [27]. It reached 90 researchers, 25 mental health professionals and 5 PhD students. Protected time and support for mental health research in clinical settings, train-the-trainers models of research education, funded study and conference leave, to develop peer networks and collaborations were key recommendations.

Little is known about the needs of early-career researchers and practitioners, to study and address GBV and its relationship to mental health in LMICs. Such knowledge will ensure that capacity-building initiatives meet the ‘felt needs’ identified by LMIC researcher and patient stakeholders [28]. WHO has recognised the requirement for the global health workforce to be “collaborative practice-ready” and recommended interprofessional education to improve responses to local health needs [29]. This pilot study aimed to identify the research, education and capacity building priorities of individuals working on violence, abuse and mental health in LMICs, and develop recommendations for action by interprofessional educational networks.

The international violence, abuse and mental health network (iVAMHN) was founded to fill a gap in support for LMIC early-career researchers interested in the intersection of GBV and mental health. iVAMHN is nested within VAMHN, a King’s College London (KCL)-led network which brings together UK-based stakeholders from a range of sectors [30]. The current membership comprises 53 individuals, of whom 26% (*n* = 14) are based in a LMIC (Ethiopia, India, Somaliland, South Africa, Uganda, Zambia, Zimbabwe). HIC-based members conduct research in these countries, plus Afghanistan, Bangladesh, Palestine, Sri Lanka and Vietnam. Members work predominantly in research and voluntary sector posts.

iVAMHN aims to establish and develop a network of researchers investigating the interactions between GBV and mental health in LMICs, to share methods, experience and results from this field, to identify priority research questions and conduct collaborative research. Following iVAMHN’s inaugural meeting in October 2019, multidisciplinary early-career researchers agreed on the need to survey diverse stakeholders.

## Methods

### Study design

Following discussion between founding iVAMHN members, an iterative, collaborative approach to survey design, data collection and analysis was selected. Members selected a qualitative survey design to optimise the richness of data that could be collected from respondents with diverse personal and professional experience of the subject.

### Instrument

We devised an anonymous online pilot survey aimed at researchers at any career stage, students, practitioners, voluntary sector workers and people with lived experience of violence, abuse and mental health problems. RK prepared a first draft, based on the initial discussion, which was edited by UB based on experience of survey design in India and her perspective as a potential end user of the instrument. The draft survey questions were shared with three senior researchers in this field with experience of survey design in LMICs and HICs and adapted in response to feedback. ON and FM, as early-career researchers in this field, pilot tested the survey to confirm clarity and suitability.

The survey instrument (Supplementary file 1) comprised four sections containing a total of 16 questions, including brief demographic questions about professional role, anonymised organisational affiliation, country or countries of work experience and employment. Qualitative questions sought free-text responses about research, education and capacity building priorities in the field of GBV and mental health in LMICs.

### Data collection

iVAMHN members proposed an online web link as the most accessible means of circulating the survey among individuals and groups identified as stakeholders. Data were collected using Google Forms and we circulated the qualitative survey website to networks of interested research, clinical and voluntary sector stakeholders by email, WhatsApp and word-of-mouth, with up to two reminder emails, between November 2019 and May 2020. Although the survey was targeted at individuals self-identifying as “researchers at any stage of their career (including students), practitioners, voluntary sector workers and people with lived experience”, it was also shared online via Twitter, given that iVAMHN members use the social network to engage with peers, colleagues, stakeholders and collaborators. Survey completion was entirely voluntary.

### Ethics

We instructed respondents not to include personally identifying information and no clinical studies or patient data were involved. We confirmed through the KCL research ethics office[31] that this study constituted service evaluation (of iVAMHN) and, therefore, did not require ethical clearance from the Research Ethics Committee.

### Analysis

We used thematic analysis to identify key themes [32]. We worked in two groups, pairing more and less experienced qualitative researchers to examine responses pertaining to current practice (AB and ON) and future priorities (UB and FDM). We followed the six step approach, with the two groups first (1) familiarising themselves with the data and (2) generating initial codes. RK then (3) searched for themes by collating codes into candidate themes and (4) reviewing themes. Multidisciplinary co-authors then collaboratively reviewed themes with the most experienced team members (JM and PA) to (5) define and name them before (6) producing this report.

We used reflexivity to identify personal biases, influenced by intersectional aspects of our personal circumstances [33]. For example, as researchers based in HICs, RK, AB, PA and JM employed self-reflexivity when reading comments from respondents based in LMICs. We also used reflexivity within our analytical team when reviewing codes and themes through collaboration between colleagues with diverse experience, which influence our interpretations. We recognised the importance of this approach to enhance ‘sense-making’ within cross-cultural, collaborative research [34]. We followed Braun and Clarke’s checklist for good thematic analysis [32], to ensure rigour in our approach. Where respondents highlighted specific resources, we identified references, where available.

## Results

### Respondents

Thirty-five unique respondents completed the survey, after excluding four blank and two duplicate entries. Respondents’ roles comprised senior lecturers or above (20%; *n* = 7), post-doctoral researchers (20%; *n* = 7), health workers (11%; *n* = 4), non-government organisation (NGO) or voluntary sector practitioners (9%; *n* = 3), manager/directors (9%; *n* = 3), lecturers (9%; *n* = 3), PhD students (9%; *n* = 3), pre-doctoral researchers (9%; *n* = 3), administrators (3%; *n* = 1) and master's degree students (3%; *n* = 1).

Respondents reported experience of working in 25 LMICs (see Table 1); eight worked in more than one country. Seventy-four percent of respondents (*n* = 26) mentioned working in one of 11 sub-Saharan African countries, the commonest being South Africa (*n* = 8), Zimbabwe and Ethiopia (both *n* = 3). Forty percent (*n* = 14) of respondents mentioned working in one of eight Asian countries, the commonest being India (*n* = 6) and Sri Lanka (*n* = 2). Respondents also worked in Latin American (*n* = 3), Eastern European (*n* = 3) and Middle Eastern countries (*n* = 1). Forty percent (*n* = 14) were based or employed in the UK, 6% (*n* = 2) in Europe and 3% (*n* = 1) in the United States, with the remaining 51% (*n* = 18) based in LMICs. Respondents’ principal organisational affiliations (see Table 2) were universities (69%, *n* = 24), non-governmental organisations (17%, *n* = 6), healthcare organisations (9%, *n* = 3), international policy and non-university education institutions (3%, both *n* = 1).

**Table 1:** LMICs in which respondents had experience of working. Some respondents worked in more than one country

Region	Country	Respondent *n* (%)
Latin America	Brazil	2 (6%)
	Trinidad	1 (3%)
Eastern Europe	Romania	1 (3%)
	Moldova	1 (3%)
	North Macedonia	1 (3%)
Sub-Saharan Africa, not specified		1 (3%)
West Africa	Nigeria	2 (6%)
	Ghana	1 (3%)
East Africa	Somalia, Somaliland	2 (6%)
	Ethiopia	3 (9%)
	Kenya	1 (3%)
	Tanzania	2 (6%)
	Uganda	2 (6%)
Southern Africa	Malawi	1 (3%)
	Zambia	1 (3%)
	Zimbabwe	3 (9%)
	South Africa	8 (23%)
Middle East	Palestine	1 (3%)
Western Asia	Iraq	1 (3%)
Central Asia	Kazakhstan	1 (3%)
South Asia	India	6 (17%)
	Sri Lanka	2 (6%)
	Nepal	1 (3%)
South-East Asia	Thailand	1 (3%)
	Malaysia	1 (3%)
	Philippines	1 (3%)

**Table 2:** Respondents’ principal organisational affiliation

Organisation	Respondent *n* (%)
University	24 (69%)
Non-government or voluntary sector organisation	6 (17%)
Healthcare organisation	3 (9%)
International policy organisation	1 (3%)
Non-university educational institution	1 (3%)

Over-arching themes addressed current work on GBV and mental health in LMICs (supplementary file 2), barriers to researching this field, how to address them, research priorities and recommendations for networks to build capacity.

### Barriers to research

Respondents identified staffing, funding, resource, sensitivity, gender norm and buy-in barriers to researching GBV and mental health in LMICs. High workforce turnover in services best-placed to address GBV and mental health and a lack of training in research design and conduct among front-line staff creates challenges. The tradition of providing short-term funding for brief projects in LMICs is an obstacle to longitudinal studies investigating causal relationships. Several respondents highlighted “extensive and difficult” ethical approval processes as barriers, “especially with vulnerable populations like children, adolescents and pregnant women”.

Practical barriers to conducting rigorous research in this field include the lack of open access metrics of violence exposure validated for LMICs, insufficient experienced, skilled interviewers to conduct qualitative research in some settings, and difficulty speaking to survivors privately, where “often family members will be present, which inhibits reporting of abuse”.

Cultural sensitivity and “taboo/inhibited responses” to asking about GBV and mental health and lack of awareness of their inter-relationship are barriers to researching them:

Women simply do not report their [domestic violence] cases because culturally, they should not share what happens in their marriage. Also, poverty forces most abused women to stay in abusive homes due to financial dependency.

Health worker, Zambia

GBV is often normalised or stigmatised, influencing people’s ability to disclose. A researcher from Zimbabwe said:

Among the clients who access [mental health] services there are some who report GBV but most of them do not have knowledge about the abuse, hence this kind of violence is considered as “normal violence” by the victims and the communities.

A UK-based health worker said:

People are generally silenced through violence and abuse. The stigma it carries… The lack of support [for] following up on mental health [problems]. Why would someone say exactly what they’re going through and feeling? This all impacts on the research being done.

Respondents highlighted barriers to recruiting diverse participants, raising:

The challenges of getting an intersectional approach, particularly given that stigma of people with mental health needs is an issue.

Policy Fellow, UK

A service manager from South Africa said that “prevailing patriarchal beliefs among local practitioners, researchers and senior health and social development officials” obstruct research progress. A post-doctoral researcher based in India highlighted that the risk of identifying GBV and mental health problems during research requires:

…supportive and trusting relationships with organisations working with these women and who would be able to continue supporting them once the research has done. But these research relationships need to be beneficial and not harmful to the organisation or the women, and this can take time to build (which we don't always have).

A senior researcher argued that:

The current generation of mental health leaders… do not know the special considerations of gender and violence and thus tend to overlook it in their programming. Yet, much of the funding goes to [them] and it’s tricky to break through as a younger researcher.

A range of practical, operational, ideological and leadership barriers was reported to hinder progress in researching the intersection between GBV and mental health in LMICs.

### Addressing barriers

Respondents argued that addressing barriers is vital for “building the next generation of mental health researchers that have a theoretical and practical background in violence”. Recommendations included practical tools to facilitate research studies, fostering collaboration, GBV training and integrating trauma-informed perspectives across research and clinical care.

To build research capacity, respondents recommended a minimum outcome set for GBV and mental health research, to standardise their measurement across different LMICs. Standardised safety protocols that support GBV survivors must be integrated more uniformly into research designs, including appropriate reporting of abuse disclosures.

The need to strengthen collaborative approaches to co-produce research with mental health service users and GBV survivors in LMICs was highlighted. Bridges need to be built between health systems and services addressing social determinants of health:

We need much more awareness of social work strategies to tackle violence outside of the health system… and much more dialogue with non-health actors who are working on these issues.

Postdoctoral researcher, Latin America, Africa, Asia

Respondents recommended widespread, regular academic and practical training for “practitioners, researchers and officials” on asking about and responding to GBV. Training should address topics of “gender, human rights and values clarification”, to encourage prioritisation of GBV and its mental health consequences at the highest levels:

Build productive relationships with ministries of health and other relevant stakeholders… to encourage collaborative, multi-disciplinary research and responses. Top-down encouragement of collaboration may be necessary where repeated attempts at grassroots efforts have failed to address deep-rooted resistance.

Postdoctoral researcher, Europe, Asia

Engage key stakeholders early on, get buy in from policy makers and key influencers in the fields of health and research to help shift the conversation.

Predoctoral researcher, Latin America, Middle East, Asia

Specific training [35] was recommended for staff working in LMIC humanitarian settings. Such training should address:

Provision of psychological first aid to [violence] survivors… better education on recognition of IPV and follow up in humanitarian settings for front-line health providers.

Predoctoral researcher, Latin America, Africa, Asia

Respondents emphasised the importance of trauma-informed approaches. They highlighted the intersection of GBV with subsequent traumatic experiences of mental healthcare in LMICs:

How to avoid re-traumatising people through coercive practices within the mental health system (e.g. QualityRights initiatives)? And we need an understanding of the particular needs of vulnerable groups, such as people with severe mental illness, to inform support systems and care.

Postdoctoral researcher, Latin America, Africa, Asia

Vicarious trauma [36] experienced by staff working with GBV survivors and the need to support their self-care [37] were also emphasised.

### Research priorities

Research priorities comprised contextual understanding of the intersection of GBV and mental ill-health in specific settings and evidence-based interventions to address them. Several respondents raised the aetiology of violence and abuse and how they impact mental health in different contexts:

Understanding the complex cultural/political/social/psychological and biological factors that lead to perpetration of violence (often by men) and thus identifying preventative strategies.

Senior researcher, Malawi

A post-doctoral researcher contended that:

The case that violence/abuse are important determinants of mental health is pretty strong already. What we’re missing is evidence of whether interventions that target violence/abuse can reduce mental health problems, which would be a powerful advocacy tool.

A majority of respondents prioritised research into effective interventions for different settings, especially those delivered by non-specialists or survivors through task-sharing approaches:

What are the common components of… evidence-based programmes that address both violence, abuse, and mental health? …For whom [are they effective]? What is the extent of scale up of these programmes?

Postdoctoral researcher, Europe, Africa, Asia

What is the effectiveness of non-specialised health worker delivery of mental health interventions for violence survivors? What is the impact of survivor-led interventions in post-conflict settings?

Manager/Director, Uganda

Overall, research priorities focused on characterising the relationship between GBV and mental ill-health in different LMIC contexts and developing feasible interventions to address them.

### Network benefits

Respondents proposed three benefits of networks for violence, abuse and mental health research in LMICs. First, networks should facilitate knowledge and cross-cultural exchange, through visits to different research settings, and capacity building, by sharing educational and methodological resources. Second, they should foster collaboration, bringing members together for grant funding applications, training and advocacy. Third, networks like iVAMHN should research practical solutions, such as counselling skills training for health workers, or community awareness programmes about GBV and mental health.

In terms of exchange, respondents proposed exchanging learning from practice and research experience, sharing evaluation tools, good practice examples and disseminating findings. They proposed sharing datasets, to clarify the prevalence and risk factors for GBV and mental ill-health in different settings and tailor prevention and treatment interventions. Shared resources could include training materials, study protocols, freely available measurement tools, intervention manuals and open access publications. An interactive online repository of innovative programmes, alongside summaries of the evidence base [38] was suggested.

Respondents envisaged networks as incubation centres for new interventions, research strategies and practical policies. They emphasised the benefits to LMIC researchers of learning from peers’ work and experiences, staying up-to-date, reducing duplication and increasing study replication. Respondents were interested in hearing members’ views in response to this survey. They advocated network activities influencing real-world practice:

We should move away from… recommendations for practice…mostly read by other academics … [we need] a collective action process, in which DV centres in different institutions work together and in close collaborations with services and stakeholders, to produce actual change in DV practices.

Lecturer, UK

Respondents also emphasised mutual advantages to network members of sharing learning:

There could be significant benefits to LMIC researchers of collaborating and sharing resources with colleagues in HICs, with mutual benefits of knowledge and experience for HIC researchers.

PhD student, Ethiopia

Facilitating knowledge and cross-cultural exchange, capacity building, fostering collaboration and conducting research are potential benefits afforded by networks like iVAMHN.

### Accessibility

Respondents emphasised the need to make network activities as accessible as possible. They proposed in-person network events hosted by members in different LMICs, with organisation shared between institutions and online access for those unable to travel or participate synchronously. “Clear and accessible communication channels”, online networking, sharing educational and research events and open access media, through a website, newsletters and social media, were recommended. Respondents proposed that networks should provide LMIC researcher bursaries and advocate for reduced and free conference places where possible. Only 29% (*n* = 10) were able to access funding to attend in-person events. Respondents emphasised the need to invest in LMIC early-career researchers, link them to work and research opportunities, and promote the field through editorials and other media.

### Activities

A broad range of network activities was recommended, focused on disseminating knowledge and resources. These included online conferences, webinars and podcasts to ensure far-reaching, accessible learning, to capacity-build and up-skill members.

Respondents proposed that through networks, small groups of LMIC- and HIC-based members could share expertise and co-apply for competitive research grant funding. This could include co-production between researchers, GBV survivors and mental health service users, adhering to principles of participatory methods and prioritising ethics and safety.

Collaborative research suggestions ranged from epidemiological studies to intervention development and implementation. Ideas included uniform needs assessments and comparisons of GBV and mental health in different LMICs, capturing health worker perspectives, validating violence measurement tools in LMICs, incorporating mental health outcomes into violence intervention evaluations and evaluating survivor-led mental health interventions. Respondents highlighted the need for interventions tailored to the specific context.

The importance of multi-country and interdisciplinary collaborations was emphasised. Respondents proposed building links with larger networks, such as the African Alliance for Maternal Mental Health [39] and the International Marcé Society for Perinatal Mental Health [40], as well as LMIC violence practitioners, faith-based organisations and men’s groups. They proposed that a cohort of champions could influence change at regional and national levels and develop local links, to facilitate research and overcome administrative and ideological barriers.

## Discussion

Whilst Delphi [22, 41] and other consensus-based studies [21, 23] canvas expert opinion on a large scale, they often address single subjects (such as mental health or violence) and prioritise broad research questions without addressing the pragmatic concerns of practitioners on the ground, especially in resource-limited settings. Our pilot study of 35 professionals and students engaged in violence, abuse and mental health research in LMICs focused on the immediate, practical priorities of stakeholders.

Stakeholder-identified priority research questions on violence, abuse and mental health in LMICs include their aetiology in different contexts and which task-shared interventions are effective where, and for whom [42]. Respondents to this pilot survey were actively involved in a range of research projects, with particular focus on perinatal women, children and adolescents, people living with HIV and refugee populations. Research priorities include more GBV and mental health research among men, women outside the perinatal period and participants experiencing intersectional gender and other disadvantages in LMICs. Educational priorities include addressing sensitivity towards asking about GBV, responding to the normalisation and stigmatisation of GBV, and to patriarchal attitudes among decision-makers and senior staff. Capacity building priorities include funding, interviewer training, streamlined ethical approval processes, provision of open-access violence metrics validated for LMICs and support services for GBV survivors following disclosure.

Research priorities identified by stakeholders in this pilot survey are in keeping with those of high level commissions linking social determinants to health outcomes in LMICs [43]. However, they also highlight subjects more likely to be neglected in LMICs, such as the needs of women outside the perinatal period or of those experiencing intersectional disadvantages. Some topics, such as the aetiology of violence in LMICs, have been well-studied by demographic and health surveys (DHS) [44–46] but their inter-relationship with mental health remains under-studied.

Indeed, despite our goal to explore the interface between mental health and GBV, most responses focused more on either mental health or GBV. Research into syndemics (synergistic epidemics: when disease states interact detrimentally[47]) has shown the health impacts of interacting social, economic, environmental and political inequality. For example, the cumulative impact of substance abuse, violence and HIV in the US was exacerbated by poor housing, poverty, stigma and lack of social support [48]. Interprofessional early-career networks [29] can promote new evidence and encourage intersectional approaches. Publicising open access resources (such as the searchable DHS platform [49]), to facilitate work at the intersection of mental health and GBV is another important function, since much peer-reviewed literature remains inaccessible without subscription.

Education priorities focused predominantly on the practicalities of conducting research into sensitive subjects in LMICs. Interestingly, these sensitivity challenges shared some commonalities with established barriers to screening for [50] or identifying GBV in HICs [51]. These include concerns about angering or offending the person, inadequate resources to support survivors once identified, confidence, gender and cultural barriers. Capacity building priorities emphasised logistical barriers to combining clinical and academic work in LMICs [27] but also highlighted key resources and training investments required to facilitate GBV research by non-specialists in low-resource settings.

How might stakeholders’ priorities be addressed? The widespread uptake of WHO’s mental health gap action programme (mhGAP) intervention guide [52] demonstrates the power of international organisations to focus national attention on stigmatised subjects. Our survey indicates the breadth of interest in GBV and its mental health impacts among diverse stakeholders. Evidence suggests that institutional partnerships that build trusting working relationships over years have the potential to overcome cultural barriers to addressing sensitive subjects such as gender inequality [53].

Other GBV and mental health priorities could be advanced by delivering WHO training [54] to all health and policy staff, including in humanitarian settings, addressing human rights in mental healthcare [55], closer collaboration with NGOs and building research capacity, including among GBV survivors and mental health service users. Research could be more efficient if minimum outcome sets, safety protocols, training and educational resources could be shared across networks like iVAMHN. Preventing and addressing vicarious trauma is particularly important for safeguarding staff wellbeing, reducing workforce turnover and limiting burnout [37].

NGOs are delivering advocacy, empowerment, parenting, violence prevention and health worker response interventions, with potential for collaboration between academia and the voluntary sector on implementation research. Respondents praised WHO guidance and policy for raising the profile of GBV [56], but emphasised the need to implement training curriculums on a large scale.

Networks like iVAMHN have the potential to connect LMIC and HIC practitioners through mutual knowledge sharing and collaboration. Many barriers in this field operate at systems levels (such as funding) or societal levels (such as normalisation of violence), outside the scope of such networks. iVAMHN and others can, however, encourage early-career researchers and practitioners to support each other to problem solve, through a community of practice [57]. Recommended activities include disseminating opportunities and educational resources, holding affordable in-person events in rotating LMICs where feasible, with online access where unfeasible, collating research, building links with relevant international organisations, and bringing research institutions together with GBV survivors and mental health service users for co-production and collaborative grant applications.

The benefits of widening access to practically relevant research and implementation materials in a timely manner are clear. Delays in the publication of research findings [58] and publication bias [59] are barriers to rapid scale-up of effective interventions in LMICs. Whilst greater targeted investment in LMIC early-career researchers [26] is needed, networks like iVAMHN have the potential to offer more accessible benefits to larger numbers of stakeholders.

### Limitations

Our pilot study’s pragmatic nature meant that the respondent sample could not be representative of the full diversity of stakeholders, especially GBV survivors and mental health service users in LMICs. Attention to the perspectives of stakeholders with lived experience and others not captured by our pilot study is a priority, using more in-depth research methods, such as qualitative interviews and focus group discussions. A strength of our study was to gather perspectives from diverse participants but priorities for research, education and capacity building in this field would benefit from dedicated qualitative research to examine them individually, with expert stakeholder groups. We captured expertise from specific centres, but the perspectives of stakeholders from other countries require investigation. Almost half of our sample was employed in a HIC, influencing the range of experiences reflected.

Our study’s timing, with most responses submitted prior to the COVID-19 pandemic, meant that recommendations such as in-person meetings cannot currently be safely implemented. Widespread uptake of online teleconferencing and online streaming is promising, where technological resources and internet connectivity allow. However, remote networking has social and practical limitations, and the benefits to early-career researchers of in-person interaction persist [60]. International observations suggest increased help-seeking for GBV [61] during COVID-19 lockdowns [62], making research in this field, when safe [63], an important priority..

### Supplementary Information

Below is the link to the electronic supplementary material.

Supplementary file1 (DOCX 26 kb)

## Data Availability

Anonymised data can be made available upon reasonable request.

## Code availability

No code was used in this study.
