# Supplementary material for: Research, education and capacity building priorities for violence, abuse and mental health in low- and middle-income countries: an international qualitative survey
Source: Soc Psychiatry Psychiatr Epidemiol. 2021 Mar 25;58(12):1761–71. doi: 10.1007/s00127-021-02061-5 (PMC10627995; doi:10.1007/s00127-021-02061-5)
Supplement: Supplementary file 1 — Supplementary file1 (DOCX 26 kb) [file 127_2021_2061_MOESM1_ESM.docx]

### Supplementary material 2: Current work on GBV and mental health in LMICs

### Current Work

Respondents described their own and others’ work on GBV and mental health in LMICs. These comprised research, intervention implementation, training and guidance, although examples often cut across multiple themes.

#### Research

##### Violence-focused Research

Respondents described GBV-focused research in LMICs using diverse methodological approaches. These included observational studies of local violence strategies, training to build health worker capacity to respond to GBV, an IPV-reduction trial for female sex workers in India[1] and a longitudinal study of intergenerational violence transmission in South Africa.[2]

The UK-funded ‘what works to prevent violence against women and girls’[3] programme was highlighted, which evaluates the social and economic costs of violence in conflict and humanitarian crisis settings. In Uganda, a randomised controlled trial (RCT) sought to reduce physical violence by primary school staff towards pupils.[4,5] In South America, one study investigated gender and health inequalities in Peru using a structural violence framework.[6] Another used mixed methods to study whether cash and food transfers could reduce IPV in Ecuador.[7]

##### Mental health-focused Research

Respondents described mental health-focused research investigating the population prevalence of common mental disorders and trials of task shared problem-solving therapy in Zimbabwe[8] and Ethiopia,[9] and online approaches in Uganda.[10] Perinatal mental health was a particular focus in South Africa and Ethiopia. An NGO worker from Zimbabwe said:

My main role is to provide problem solving therapy to individuals at primary health care level so as to increase mental health access and awareness to every individual at no cost, with the aim to empower people to be independent and confident enough to face their challenges and come up with viable solutions that make life worthwhile for them. I also assist with mental health research, assessing impact of our intervention in various disciplines and trying to come up with various intervention methods that suit diverse individuals.

##### Violence and Mental Health Research

Most research studies mentioned by respondents addressed the intersection between mental health and GBV, predominantly in sub-Saharan Africa. Methodological approaches included RCTs, epidemiological studies and mixed methods. Most research focused on mental health interventions and mental health outcomes in survivors of violence. A researcher working in India described:

Investigating the effectiveness of mental health interventions in improving the functioning and wellbeing of women who have experienced violence with a view to developing interventions that are suited to use in LMIC settings.

One respondent described an RCT evaluating the impact of community mobilisation on the prevalence of GBV in Mumbai, with mental health as a secondary outcome. Another cited a mental healthcare package being developed for survivors of multiple forms of violence in India, Sri Lanka and Afghanistan.[11] Specific interventions included one addressing IPV and psychological distress in a Tanzanian refugee camp [12] and cognitive processing therapy for survivors of sexual violence in Eastern Democratic Republic of Congo.[13]

Other research addressed the intersection of GBV, mental health and HIV. An RCT is studying their relationship with immunity in female sex workers in Nairobi.[14] A South Africa-UK university collaboration is exploring suicidality, violence exposure and anti-retroviral therapy adherence in adolescents living with HIV.[15,16] Other South African studies are assessing IPV and mental health in HIV-affected adolescent parents,[17] the impact of trauma-focused CBT on preventing mother-to-child HIV transmission[18] and conducting mixed methods research.[19]

#### Intervention Implementation

##### Violence interventions

Respondents from a mixture of NGOs, universities, healthcare and international policy organisations described interventions focused on violence and abuse, including domestic violence and violence against children. Advocacy interventions providing platforms for dialogue or raising awareness of GBV were described in Uganda and South Africa. An NGO worker from South Africa described work to:

Explore ways to find local solutions to address gender based violence, to explore meaningful ways to engage all genders to those dialogues and to implement programmes addressing the above.

Some respondents were engaged in designing or developing violence interventions in LMICs, including healthcare interventions and parenting programmes.[20] Others are implementing interventions to prevent violence and abuse. One international policy organisation builds the capacity of health workers to respond to GBV.

##### Mental health interventions

Respondents had developed district mental health plans and community mental health networks in refugee settlements. Specific interventions in refugee communities included advocacy, such as empowerment through enhanced problem solving skills, and sharing experiences via social media platforms. In Uganda, lay health workers or primary care staff deliver psychological first aid and facilitated groups; some target children and adolescents. One NGO focused on the wellbeing of Indian farmers, including suicide prevention and women’s empowerment.[21] The Mental Health Innovation Network (MHIN)[22] was highlighted, which supports the development and uptake of effective mental health interventions, by facilitating education, partnership, knowledge and resource sharing.

##### Violence and Mental Health interventions

Interventions focused on both violence and mental health included parenting programmes to reduce violence against children and its mental health consequences, programmes to prevent bullying and its mental health impacts, advocacy interventions to increase awareness of GBV and mental health in university students, and health worker training. Most interventions addressing both violence and mental health were implemented in Sub-Saharan Africa, including Zimbabwe, Ethiopia, South Africa, Ghana, Nigeria and Tanzania. An NGO worker from Zimbabwe said:

I work as a coordinator of a mental health organization which provide services for clients experiencing common mental disorders…We are simply encouraging health care givers to talk about dangers associated with this kind of abuse but nothing much has been put in place to address this topic.

Specific services included free mental healthcare for survivors in urban Zambia,[23] where a health worker said: We are currently running a project through which we provide free mental health services to women and girls who are survivors of sexual and gender-based violence in poor urban areas.

#### Training and guidance

Respondents praised guidance and curriculums on violence and mental health produced by WHO. These included ethical and safety recommendations for research on violence against women,[24,25] a new curriculum to equip health workers to respond to GBV[26] and a recently-published guide on post-rape care in humanitarian settings.[27] Other innovative work includes a World Psychiatry Association competency-based curriculum[28] and online GBV training for health workers.[29] WHO’s ‘QualityRights’ initiative, improving the quality of mental healthcare through a rights-based and recovery-oriented approach, was also praised.[30]

South African universities have developed health worker training and digital resources on GBV and maternal mental health. An NGO staff member working in Uganda described how:

We take a partnership approach to education and capacity strengthening in our research group, encouraging colleagues from all country teams, and also ECR [early career researcher] colleagues to take the lead on delivering certain aspects of training.

Although respondents were involved in and aware of a breadth of research, interventions, training and guidance focused on GBV and mental health in LMICs, these were affected by a range of barriers.

### References

1. Javalkar P, Platt L, Prakash R, Beattie TS, Collumbien M, Gafos M, Ramanaik S, Davey C, Jewkes R, Watts C (2019) Effectiveness of a multilevel intervention to reduce violence and increase condom use in intimate partnerships among female sex workers: cluster randomised controlled trial in Karnataka, India. BMJ Global Health 4 (6)

2. The_University_of_Edinburgh (2019) ERC funds Social Work team’s study on intergenerational violence in South Africa. <http://www.sps.ed.ac.uk/about/news/2019/erc_funds_social_work_teams_study_on_intergenerational_violence_in_south_africa>. Accessed 17/10/2020

3. What_Works (2019) About What Works. <https://www.whatworks.co.za/about/about-what-works>. Accessed 17/10/2020

4. Devries KM, Knight L, Child JC, Mirembe A, Nakuti J, Jones R, Sturgess J, Allen E, Kyegombe N, Parkes J (2015) The Good School Toolkit for reducing physical violence from school staff to primary school students: a cluster-randomised controlled trial in Uganda. The Lancet Global Health 3 (7):e378-e386

5. Raising_Voices (2020) Good School Toolkit. <https://raisingvoices.org/good-school/>. Accessed 17/10/2020

6. Shannon GD, Motta A, Cáceres CF, Skordis-Worrall J, Bowie D, Prost A (2017) ¿Somos iguales? Using a structural violence framework to understand gender and health inequities from an intersectional perspective in the Peruvian Amazon. Global Health Action 10 (sup2):1330458

7. Buller AM, Hidrobo M, Peterman A, Heise L (2016) The way to a man’s heart is through his stomach?: a mixed methods study on causal mechanisms through which cash and in-kind food transfers decreased intimate partner violence. BMC Public Health 16 (1):488

8. Chibanda D, Weiss HA, Verhey R, Simms V, Munjoma R, Rusakaniko S, Chingono A, Munetsi E, Bere T, Manda E (2016) Effect of a primary care–based psychological intervention on symptoms of common mental disorders in Zimbabwe: a randomized clinical trial. JAMA 316 (24):2618-2626

9. Keynejad RC, Bitew T, Sorsdahl K, Myers B, Honikman S, Medhin G, Deyessa N, Sevdalis N, Tol WA, Howard L (2020) Problem solving therapy (PST) tailored for intimate partner violence (IPV) versus standard PST and enhanced usual care for pregnant women experiencing IPV in rural Ethiopia: protocol for a randomised controlled feasibility trial. Trials 21:1-15

10. Uzima_Ari (2020) Uzima Ari Uganda.

11. UCL (2020) A package of care for the mental health of survivors of violence in South Asia. <https://www.ucl.ac.uk/global-health/research/z-research/package-care-mental-health-survivors-violence-south-asia>. Accessed 17/10/2020

12. Greene MC, Rees S, Likindikoki S, Bonz AG, Joscelyne A, Kaysen D, Nixon RD, Njau T, Tankink MT, Tiwari A (2019) Developing an integrated intervention to address intimate partner violence and psychological distress in Congolese refugee women in Tanzania. Conflict and Health 13 (1):38

13. Resick PA, Williams LF, Suvak MK, Monson CM, Gradus JL (2012) Long-term outcomes of cognitive–behavioral treatments for posttraumatic stress disorder among female rape survivors. Journal of Consulting and Clinical Psychology 80 (2):201

14. London_School_of_Hygiene_and_Tropical_Medicine (2020) Maisha Fiti. <https://www.lshtm.ac.uk/research/centres-projects-groups/maisha-fiti#welcome>. Accessed 17/10/2020

15. Cluver L, Meinck F, Toska E, Orkin FM, Hodes R, Sherr L (2018) Multitype violence exposures and adolescent antiretroviral nonadherence in South Africa. AIDS 32 (8):975

16. Casale M, Boyes M, Pantelic M, Toska E, Cluver L (2019) Suicidal thoughts and behaviour among South African adolescents living with HIV: Can social support buffer the impact of stigma? Journal of Affective Disorders 245:82-90

17. Young_Carers_SA (2015) Hey Baby. <http://www.youngcarers.org.za/hey-baby>. Accessed 17/10/2020

18. Hatcher AM, Woollett N (2020) Project Brief: Relationship safety and mental health for HIV-positive pregnant women. <https://www.researchgate.net/publication/344738851_Project_Brief_Relationship_safety_and_mental_health_for_HIV-positive_pregnant_women>. Accessed 19/10/2020

19. Mzantsi_Wakho (2020) About Mzantsi Wakho. <http://www.mzantsiwakho.org.za/about>. Accessed 17/10/2020

20. University_of_Oxford (2020) Parenting for Lifelong Health SUPER (Scale-Up of Parenting Evaluation Research) Study. <https://www.spi.ox.ac.uk/parenting-for-lifelong-health-SUPER>. Accessed 17/10/2020

21. Dilasa (2020) About us. <http://www.dilasa.org/>. Accessed 17/10/2020

22. MHIN (2014) About. <https://www.mhinnovation.net/about>. Accessed 17/10/2020

23. PsycHealth_Zambia (2020) How we bring change. <https://psychzambia.com/>. Accessed 18/10/2020

24. WHO (2016) Ethical and safety recommendations for intervention research on violence against women. <https://apps.who.int/iris/bitstream/handle/10665/251759/9789241510189-eng.pdf;jsessionid=CEA6AEFEB81817CF0C3AF44106773CDC?sequence=1>. Accessed 18/10/2020

25. Ellsberg M, Heise L (2005) Researching violence against women: a practical guide for researchers and activists. <https://path.azureedge.net/media/documents/GBV_rvaw_complete.pdf>. Accessed 18/10/2020

26. WHO (2019) Caring for women subjected to violence: A WHO curriculum for training health-care providers. <https://www.who.int/reproductivehealth/publications/caring-for-women-subject-to-violence/en/>. Accessed 18/10/2020

27. WHO (2020) Clinical management of rape and intimate partner violence survivors: Developing protocols for use in humanitarian settings. <https://www.who.int/reproductivehealth/publications/rape-survivors-humanitarian-settings/en/>. Accessed 17/10/2020

28. Stewart DE, Chandra PS (2017) WPA International Competency‐Based Curriculum for Mental Health Providers on Intimate Partner Violence and Sexual Violence Against Women. World Psychiatry 16 (2):223

29. Decker M (2020) Confronting Gender Based Violence: Global Lessons for Healthcare Workers. <https://www.coursera.org/learn/gender-based-violence>. Accessed 17/10/2020

30. WHO (2019) WHO QualityRights initiative – improving quality, promoting human rights. <https://www.who.int/mental_health/policy/quality_rights/en/>. Accessed 17/10/2020
